# Automatic Roadside Feature Detection Based on Lidar Road Cross Section Images

**DOI:** 10.3390/s22155510

**Published:** 2022-07-23

**Authors:** Ivan Brkić, Mario Miler, Marko Ševrović, Damir Medak

**Affiliations:** 1 Department of Geoinformatics, Faculty of Geodesy, University of Zagreb, Kačićeva 26, 10000 Zagreb, Croatia; ibrkic@geof.unizg.hr (I.B.); dmedak@geof.unizg.hr (D.M.); 2 Department of Transport Planning, Faculty of Transport and Traffic Sciences, University of Zagreb, Vukelićeva 4, 10000 Zagreb, Croatia; msevrovic@fpz.unizg.hr

**Keywords:** Lidar, road safety, road assessment, roadside features

## Abstract

The United Nations (UN) stated that all new roads and 75% of travel time on roads must be 3+ star standard by 2030. The number of stars is determined by the International Road Assessment Program (iRAP) star rating module. It is based on 64 attributes for each road. In this paper, a framework for highly accurate and fully automatic determination of two attributes is proposed: roadside severity-object and roadside severity-distance. The framework integrates mobile Lidar point clouds with deep learning-based object detection on road cross-section images. The You Only Look Once (YOLO) network was used for object detection. Lidar data were collected by vehicle-mounted mobile Lidar for all Croatian highways. Point clouds were collected in .las format and cropped to 10 m-long segments align vehicle path. To determine both attributes, it was necessary to detect the road with high accuracy, then roadside severity-distance was determined with respect to the edge of the detected road. Each segment is finally classified into one of 13 roadside severity object classes and one of four roadside severity-distance classes. The overall accuracy of the roadside severity-object classification is 85.1%, while for the distance attribute it is 85.6%. The best average precision is achieved for safety barrier concrete class (0.98), while the worst AP is achieved for rockface class (0.72).

## 1. Introduction

According to World Health Organization (WHO), road traffic injuries are the leading cause of death of people aged 5 to 29 years [1]. Road network infrastructure is strongly linked to the consequences of road accidents and the number of fatalities [2]. Therefore, the United Nations (UN) Member States have agreed on 12 new Voluntary Global Road Safety Performance Targets to drive action across the world [3]. Two of the targets (Targets 3 and 4) include ensuring all new roads are built to a 3-star or better standard for all road users (Target 3), and that 75% of all travel is conducted on the equivalent of 3-star or better roads for all road users by 2030 (Target 4). UN estimates that 450,000 lives will be saved every year if these targets are applied in practice [4]. The number of stars is usually determined by the International Road Assessment Programme Star Rating (iRAP Star Rating). iRAP is the umbrella strategy for Road Assessment Programmes across the world (Europe—EuroRAP, Australia—AusRAP, New Zealand—KiwiRAP, China—ChinaRAP, USA—UsRAP, Brazil—BrazilRAP, South Africa—SARAP, Thailand—ThaiRAP, and India—IndiaRAP). iRAP Star Ratings is one of the five iRAP protocols, designed to collect road attributes on a particular road segment [5]. It is applicable for use also in low- and middle-income countries where data of road crashes is difficult to obtain. Likewise, iRAP Star Ratings is intended to assess infrastructure-related risk based on crash modification factors considering the likelihood and severity of individual user accidents with respect to the infrastructure features. The most dangerous roads, where the probability of a serious traffic accident with a fatal outcome is very high, are rated with 1 star, while the safest roads, where the probability of a fatal accident is zero, are rated with 5 stars [6]. In addition to the plans of the UN Global Road Safety Performance Targets, the collection of road attributes is important for European countries to comply with the European Union (EU) Directive 2019/1936 amending the 2008/96 Directive (RISM), which requires more detailed collection of road attributes to improve the safety of road infrastructure in EU member states [7]. iRAP Star Rating protocol correspond to approximately 95% of the indicative elements set out in the Annex III of the amended RISM Directive and thus can be successfully used to produce network classification in the Network-wide road safety assessment procedures.

In order to achieve the requirements of both iRAP and the EU directives there is a need for high-quality road data collection and extraction of road features. Various research efforts have approached this problem in different ways. The approaches to determining road attributes differ primarily in the selection of the sensors used to collect the data and in the selection of the techniques used to determine the attributes from the collected data. Since not all required road attributes can be automatically collected with one type of sensor, it is necessary to use different sensors to collect different attributes. For example, sensors mounted on Unmanned Aerial Vehicles (UAVs) are the most-used to collect traffic flow data [8,9,10,11]. In addition, traffic flow data can be collected using instruments such as pneumatic road tubes and induction loops [12,13,14]. When it comes to attributes related to road infrastructure, georeferenced videos [15,16] or standard videos [17,18,19] are mostly used for data collection. Stated sensors are used for data collecting, but the standard process of attribute determination is still done manually by coding attributes from the collected data [20]. For this reason, iRAP is making efforts to develop new methods for automated data collection and attribute determination by taking advantage of state-of-the-art technologies such as machine learning, telematics, and Light Detection and Ranging (Lidar) [21]. The number of studies aimed at improving data collection and attribute determination methods using these technologies is increasing [17,18,22,23].

The main objective of this study is to provide a new framework for determining road infrastructure attributes. The framework consists of a combination of mobile Lidar sensor for collecting road infrastructure data as a point cloud and deep learning techniques for object detection for final attribute determination. Both the mobile Lidar sensor and deep learning-based object detection are advanced technologies proposed by iRAP that can improve accuracy and reduce the time required to determine road infrastructure attributes. As mentioned earlier, not all attributes can be determined by a single sensor. Therefore, this paper focuses on the complete and automatic solution of only two iRAP attributes: roadside severity-object (RSS–O)—related to roadside object detection—and roadside severity-distance (RSS–D)—related to the distance of the detected object from the road edge.

The proposed framework has multiple advantages with regards to related papers and standard processes of road infrastructure attributes determination. Firstly, using Lidar sensor enables spatial consideration, which is hard to achieve from video images. The importance of spatial considerations in terms of road safety is described in [24]. Since the vast majority of iRAP RSS–O classes are defined by dimensions (length, angles, etc.) as well as the distance of an individual object from the edge of road for the RSS–D attribute, this approach improves the determination of road attributes. Secondly, this paper proposes fully automated flow for the determination of the stated attributes. It is an improvement over manual attribute coding, mostly in the significant shortening of the duration of the process, but also in the consistency of attribute determination. Thirdly, the object detection part of study enables the detection of 13 different classes specified by the iRAP Coding Manual [5] and the determination of their relative position to the road edge. Considering the complexity of iRAP RSS–O classes definitions, the classification of detected objects is achieved with high accuracy.

This paper is structured as follows: after a brief introduction to the research topic, the mention of the main contributions of this paper, and a brief overview of recent related studies, the proposed framework is described in detail. The framework is divided into two parts: data collection with point cloud processing and the object detection process on road cross section images. For better understanding of the whole process, the framework is presented with a corresponding diagram. Both parts of the framework have subsections that describe each step of the proposed framework in detail. This is followed by a results section, which presents the results of the object detection process as well as the spatial accuracy of road and roadside object detection and the final classification of road segments in terms of RSS–O and RSS–D attributes. The results are presented in the form of tables and confusion matrices. This is followed by a discussion of the results and the main advantages and disadvantages of the system in comparison with related works. Finally, based on the results and discussion, a brief conclusion is given, indicating future research options to improve the determination of iRAP attributes.

### Related Works

There are a growing number of studies investigating ways to improve and automate the process of determining road infrastructure features. While road attributes represent the characteristics of the road segment in the database, road infrastructure features represent physical objects on the road and in the roadside area. The methods differ depending on the sensor used to collect the road data and how the road attributes are extracted from the collected data. Regarding the sensors used, videos are the most used [17,18,25,26], but there are few studies that use Lidar [22,23,27].

Sanjeewani and Verma (2017) [28] proposed a Fully Convolutional Network (FCN) to automatically find all AusRAP roadside objects: lanes, poles, sign boards, trees, metal barriers, warning signs, rumble strips, guideposts, concrete medians, etc. A Fully Convolutional Network (FCN) is a neural network that only performs convolution (and subsampling or upsampling) operations. Simplified, an FCN is a Convolutional Neural Network (CNN) without fully connected layers [29]. The technique is based on vehicle-based video data extracted into frames (images). The images were divided into homogeneous regions, which were used for image segmentation into AusRAP object classes on pixel base. Segmentation is performed by automated deep learning feature extraction based on a neural network with a classifier in the last layer of the FCN. This means that one FCN was used to classify all attributes. As for the evaluation metrics, the paper reports the pixel-wise attribute classification accuracy after 10,000, 15,000, and 20,000 iterations of the FCN training procedure. Jan Z. et al. (2019) [25] proposed a CNN for the identification of all roadside objects. The technique is based on videos extracted into images. The approach is divided into three parts: image segmentation into nine AusRAP object classes by applying CNN, calculation of the distance between the road and the detected object, and evaluation of the proposed approach. As for the evaluation, a confusion matrix of the detected objects was provided, but there are no evaluation metrics for the calculated distances. In addition, the authors suggest that the use of Lidar could improve the detection results. Sanjeewani and Verma (2021) [18] improved their research from 2019, and the proposed model is also based on video data and FCN, but only one FCN is used for the detection of a single object class. Finally, an improvement is achieved by fusing all FCNs. The proposed approach is applied to 13 roadside object classes, such as speed signs, poles, trees, warning signs, etc. The paper provides an evaluation of the proposed approach with attribute-wise and pixel-wise accuracy. A comparison of the number of iterations with attribute-wise and pixel-wise accuracy is also provided. Sanjeewani and Verma (2021) [17] performed an optimization of the FCN-based approach for AusRAP attribute classification proposed in [18]. The FCN optimization is applied to four AusRAP road objects: Guidepost, Signal light, Flexipost, and Rumble strip. The optimization is based on finding the best combination of hyperparameters of the FCN, such as number of convolutional layers, activation function, pooling type, image size, number of iterations, and the optimization algorithm used. The paper also provides attribute-wise and pixel-wise accuracy for a single attribute.

When it comes to Lidar-based approaches for road data collection and determination of road attributes, Martin-Jimenez et al. (2018) [22] used mobile Lidar point clouds to assess road safety and estimate risk potential in Spain. The proposed approach is divided into four segments: classification of mobile Lidar point clouds based on geometric and radiometric properties of the point clouds; extraction of horizontal alignment and main road parameters based on geometric design consistency index; estimation of potential risk by a new predictive tool based on tree induction algorithm; and verification of the results in comparison with data from road safety experts considered as ground truth. The authors suggest that this approach may be suitable for the EuroRAP approach to risk assessment. Zhong M. et al. (2019) [23] proposed a point cloud classification framework for roadside safety attributes and distance detection. The framework consists of three stages: roadside point cloud data labeling; point cloud classification network; and object center approximation technique for distance calculation. The authors developed a system for seven roadside object classes: pole, tree, road, guard rail, sign, vehicle, and other. The object-wise accuracy and the confusion matrix are given for only two object classes—pole and tree—while the pixel-wise accuracy is given for all detected object classes. For the same two object classes, the accuracy of determining the distance to the road is also given.

Similar research has been made about road and roadside features extraction from the fusion of Lidar and images independent of iRAP standards. Ural et al. (2015) [30] proposed an approach that incorporates data from the air: Color Infrared Orthophotos and Lidar Point Clouds. They applied Support Vector Machine (SVM) to segment the road surface from orthophotos. They eliminated the main obstacles such as buildings that have color similarities with the road surface using Lidar point clouds and ground filtering based on the Tri-angular Irregular Network (TIN) compaction method. They extracted 90.25% of all studied roads. Han et al. (2017) [31] proposed a road detection method based on the fusion of Lidar and image data. First, lidar point clouds were projected onto monocular images. Then, color features were extracted from color images and used with the corresponding pixels in monocular images generated from the lidar point cloud. These data were used for pixel-wise classification of roads using Adaboost classifier. The authors achieved acceptable performance, but noted a large number of false positive road pixels. As a second limitation, they found that the number of false positive pixels increases with the increasing distance from the sensor due to the limited accuracy of the sensor. Zeybek, M. (2021) [32] propose a method for automatically extracting lane markings from lidar data. The proposed method includes many different algorithms such as Cloth Simulation Filtering (CSF) to distinguish ground and non-ground data. Moreover, Random Sample Consensus (RANSAC) method was used to filter road surface from ground points. Finally, the Canny edge operator was used to extract the contours of the lanes.

## 2. Materials and Methods

This study was conducted with mobile Lidar data from the Croatian highway network. The Croatian highway network has a length of 1306 km, i.e., 2612 km in both directions. Point clouds for the highway network in both directions form an extensive and heterogeneous basis for the process of determining road infrastructure attributes. iRAP provides a list of 64 attributes [5]. All attributes must be collected for 100 m-long segments of the observed road for the road to be rated instars from 1 to 5. Croatian highways network consists of eight main parts. Every direction of every part is considered as single road. For single road segments are created from its start point every 100 m. Furthermore, every segment is coded with appropriate codes for all of 64 iRAP attributes. As mentioned earlier, despite various research efforts on automation, the process of attribute determination for road infrastructure in practice consists of manually determining attributes for a given road segment from a georeferenced video.

**Figure 1:**
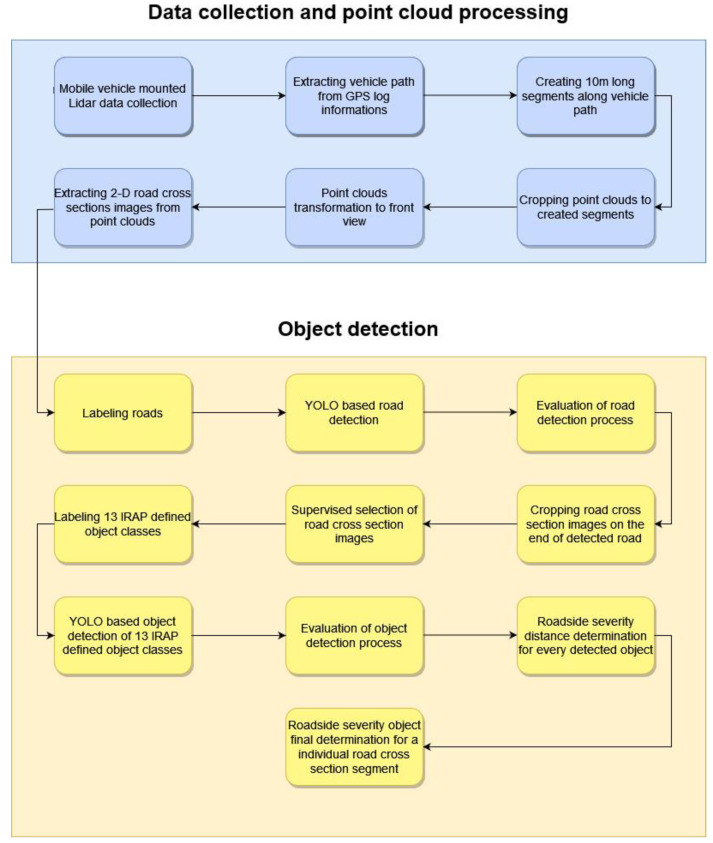
Proposed framework for the determination of RSS–O and RSS–D attribute.

The proposed framework is divided into two parts: mobile Lidar data collecting with point cloud processing and object detection process on cross-road images. Moreover, the mentioned parts are divided into more detailed parts, which are shown in Figure 1. Both parts and their corresponding subparts are explained in detail below.

### 2.1. Data Collection with Point Cloud Processing

A point cloud is a set of geometric points with coordinates sampled from 3D space [33]. Point clouds are usually generated by computer graphics or acquired by Lidar to represent 3D objects. Lidar is based on a laser that is directed at the target, and the light beam is reflected from the surface. The sensor records the reflected light to measure the distance. Combining the laser distances with data from the integrated global navigation satellite system (GNSS) and the inertial measurement system (IMU) produces a dense, detailed group of points in space, i.e., a point cloud. Data collection for the study is performed using a Trimble MX 8 Land Mobile Mapping System (Trimble, Sunnyvale, California, USA). The technical specifications of the used system are listed in Table 1.

The collected point clouds are stored in las format. Since the Mobile Mapping System has two Lidar sensors, the point clouds for each of the sensors were collected separately, so they had to be merged to obtain a higher point density and larger field of view (FOV). The merging process was performed using the Python PDAL library.

**Figure 2:**
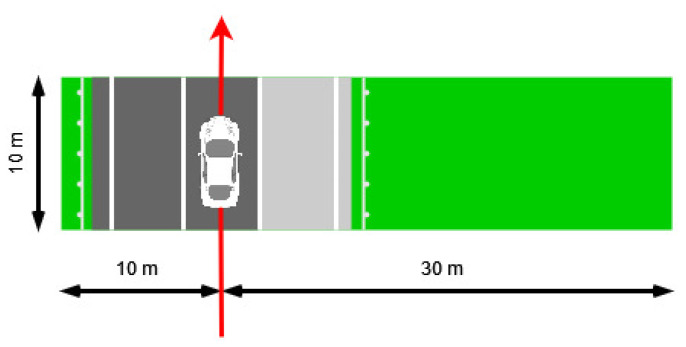
Example of one created road segment with appropriate dimensions. Red arrow represents driving direction, while black arrows represent dimensions of segment.

**Figure 3:**
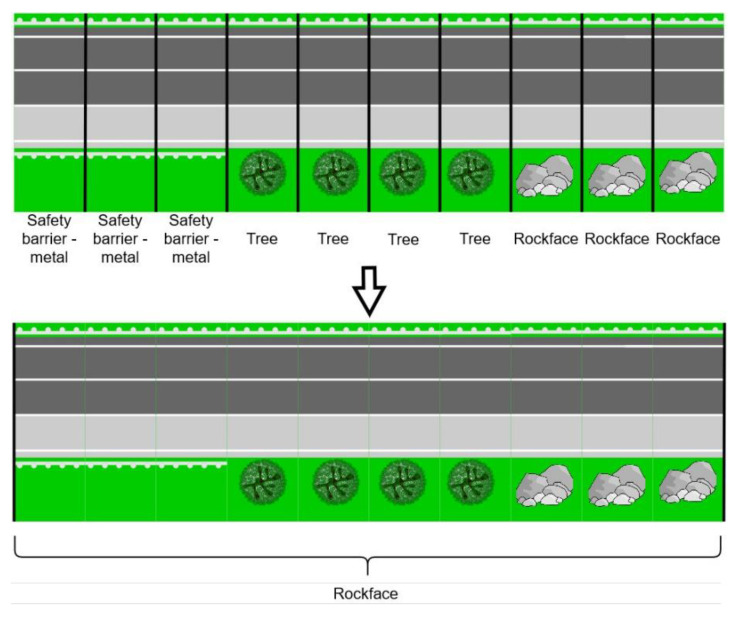
Example of upsampling 10 m road segments to one 100 m iRAP defined road segment.

Although the iRAP Manual Guide prescribes the assignment of a single object to a 100 m road segment, this paper performs this process for 10 m road segments to make more detailed determination of RSS–O and RSS–D attributes. Finally, 10 m road segments can be upsampled to 100 m road segments by selecting the most hazardous object among 10 road segments that are 10 m long. The hazardousness of an individual object is also defined in the iRAP Manual Guide by the type of roadside object and its distance from the roadside. Roadside hazards are listed in the iRAP Coding Manual [5] (page 50) in order from highest to lowest risk. The road segments are 10 m long and 40 m wide: 10 m on the left side of vehicle path and 30 m on the right side of vehicle path. The vehicle path was extracted from the Lidar GNSS log file. The data from GNSS were collected in International Terrestrial Reference Frame (ITRF) and then converted to the Croatian Reference Coordinate System (HTRS96/TM). An example of a created road segment is shown in Figure 2, while an example of upsampling 10 road segments of 10 m in length to an iRAP-defined 100 m road segment is shown in Figure 3.

After road segments were created, the collected point clouds were clipped to the boundaries of the road segments using PDAL python library.

**Figure 4:**
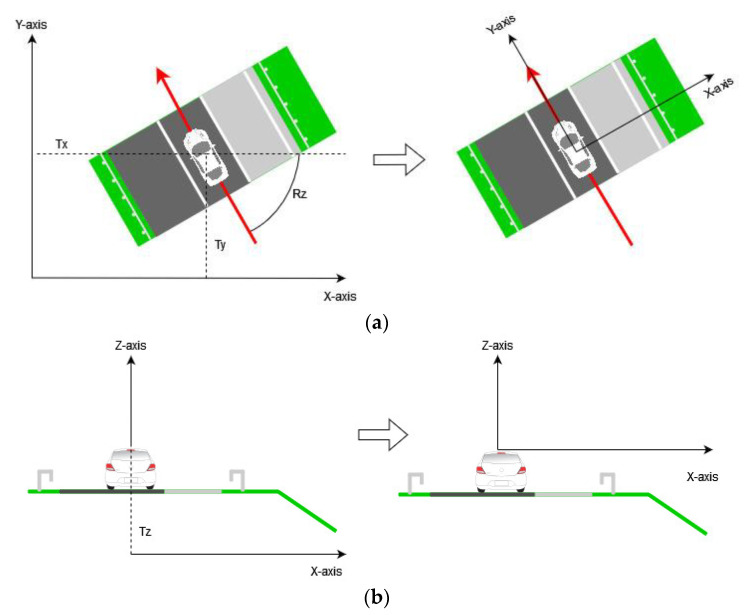
(**a**) Process of translation in direction of x-axis and y-axis and rotation around z-axis for Rz angle; (**b**) process of translation in direction of z-axis.

To perform object detection on point clouds, the detection method must be carefully selected. According to [34], there are three main methods for object detection on point clouds: projection-based methods (front view and bird’s eye view methods), voxel-based methods, and point-based methods. In this work, the frontal view method was chosen for object detection because the RSS–O and RSS-D are best distinguished from the road cross-section. In order to obtain front view images of road sections, i.e., images of road cross sections, the point clouds must be transformed from the Croatian Reference Coordinate System to a local coordinate system. The origin of the local coordinate system is at the center of the Lidar sensor, the x-axis is orthogonal to the vehicle path and the y-axis is in the direction of the vehicle path. The transformation process was performed by applying the equation:

(1) $$\left[\begin{array}{c} x^{\prime }\\ y^{\prime }\\ z^{\prime }\\ 1 \end{array}\right]=\left[\begin{array}{cccc} \cos Rz & -\sin Rz & 0 & Tx\cos Rz-Ty\sin Rz\\ \sin Rz & \cos Rz & 0 & Tx\sin Rz+Ty\cos Rz\\ 0 & 0 & 1 & Tz\\ 0 & 0 & 0 & 1 \end{array}\right]\left[\begin{array}{c} x\\ y\\ z\\ 1 \end{array}\right]\;$$

where vector [*x*′, *y*′, *z*′, 1] represents the coordinates of the single point in the point cloud after the transformation, *Tx* represents the translation in the x-axis direction, *Ty* represents the translation in the y-axis direction, *Tz* represents the translation in the z-axis direction, *Rz* represents the rotation angle about the z-axis, and the vector [*x*, *y*, *z*, 1] represents the coordinates of the single point in the point cloud before the transformation. The transformation process is shown in Figure 4.

**Figure 5:**
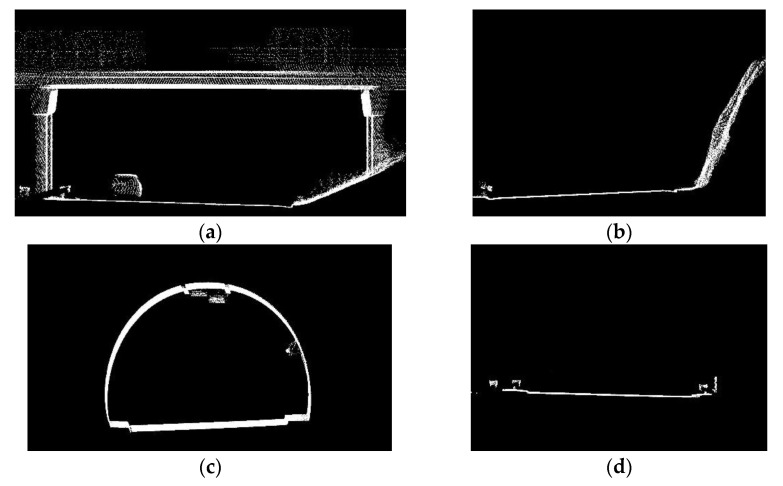
(**a**) Example of road cross section on part of road with overpass; (**b**) example of road cross section on part of road with irregular rockface; (**c**) example of road cross section on part of road with tunnel; (**d**) example of road cross section on part of road with both safety barriers.

After the transformation process, the PDAL library was used to export point clouds into orthophoto images with depth of 10 m, i.e., road cross sections for 10 m of road. The exported images have a band with values of reflectance. The spatial resolution of the exported images is 1 cm × 1 cm. In terms of height, a range of 15 m above and 10 m below the Lidar sensor is covered. According to the height profile, the dimensions of the road segments and the spatial resolution, the dimensions of the images are 2500 px × 4000 px (2500 px × 1 cm = 25 m; 4000 px × 1 cm = 40 m). Examples of four exported road cross section images are shown in Figure 5.

### 2.2. Object Detection

For the RSS–O attribute, iRAP defines 17 classes of objects, 13 of which are found on Croatian highways. The definitions of the individual object classes are given in the iRAP coding manual [5] (pages 52–54).

**Figure 6:**
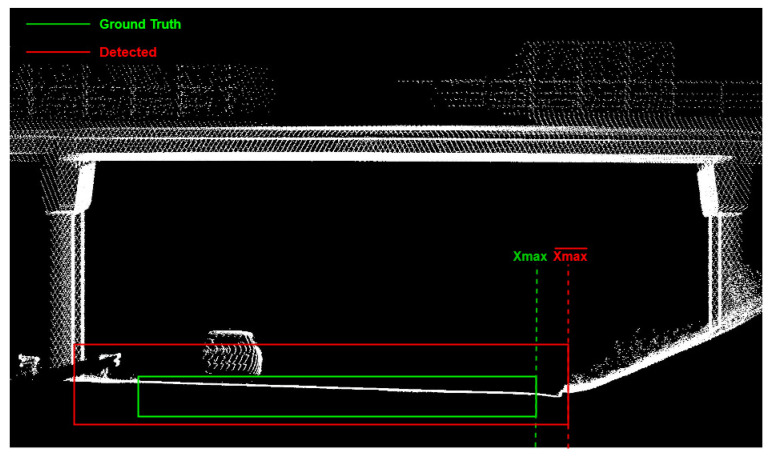
Example of road cross section with ground truth and detected road bounding boxes.

Regarding the RSS–D attribute, iRAP defines four classes: 0–1 m, 1–5 m, 5–10 m, and >10 m from the roadside. To determine the RSS–D attribute, it is necessary to determine the road bounding box, focusing on the coordinate of the right edge of the road (Xmax). An example of a road bounding box is shown in Figure 6.

Road detection is performed by deep learning-based object detection algorithm. The You Only Look Once (YOLO) algorithm was used. This is a unified model for object detection which is trained on a loss function that directly corresponds to detection performance, and the entire model is trained jointly [35]. In recent years, five versions of YOLO have been released, each time with significant changes in the algorithm structure that improved both the inference time and the accuracy of the algorithm [36]. In terms of inference time and detection accuracy, YOLO was used in this work mainly because of the need to automate the process of classifying iRAP road segments which requires a fast inference time.

As for any object detection algorithm, it is necessary to have a sufficiently large, labeled dataset that allows adequate object detection. In this study, road labeling was performed using the labeling application on 5000 images of road cross sections. The labeled road cross-section images were divided into a train and a test dataset with a 75:25 ratio, i.e., 3750 train images and 1250 test images. The training process was performed in 1000 epochs with a batch size of 2 images. The training time was 15 h and 45 min on an NVIDIA GeForce RTX 2080 Ti GPU (NVIDIA Corporate, Santa Clara, CA, USA). Prediction process results with the confidence score of each detected road and the coordinates of the road bounding box (Xmin, Ymin, Xmax, Ymax).

The mean Average Precision (mAP) is calculated to evaluate the object detection process. It is the cross-class average of the interpolated Average Precisions (AP) [37]. AP represents the area under the recall-precision curve. It is the de facto standard for evaluating object detection performance [38]. The computation of recall and precision and their meaning is described in detail in [39]. In this part of the framework, only one class (road) was detected, so mAP is equal to AP.

To evaluate the spatial accuracy of road detection, Root Mean Square Error (RMSE) value was used. RMSE is defined by equation:

$$RMSE=\sqrt{\overset{n}{\underset{i=1}{\sum }}\frac{{\left(Xmax_{i}-{\bar{Xmax}}_{i}\right)}^{2}}{n}}$$

where *n* is the number of road bounding boxes in the test dataset, *Xmax_i_* is the right road edge of a single ground truth road and $\bar{Xmax}$_i_ is the right road edge of a single detected road.

**Figure 7:**
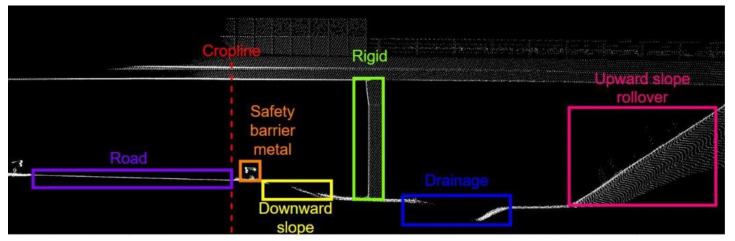
Example of road cross section with labeled iRAP defined objects.

Road cross-section images were used to label 13 iRAP-defined object classes. Supervised selection of road cross-section images was applied to label as many different object classes as possible on as few images as possible. Finally, 7804 images with 12,987 labeled objects were selected. The images were split into train dataset with 5853 images and a test dataset with 1951 images. An example of a road cross-section with labeled objects is shown in Figure 7. The training process was performed on the same graphics processor as the road detection. The training time was 35 h and 30 min with a batch size of 2 and within 350 epochs.

**Figure 8:**
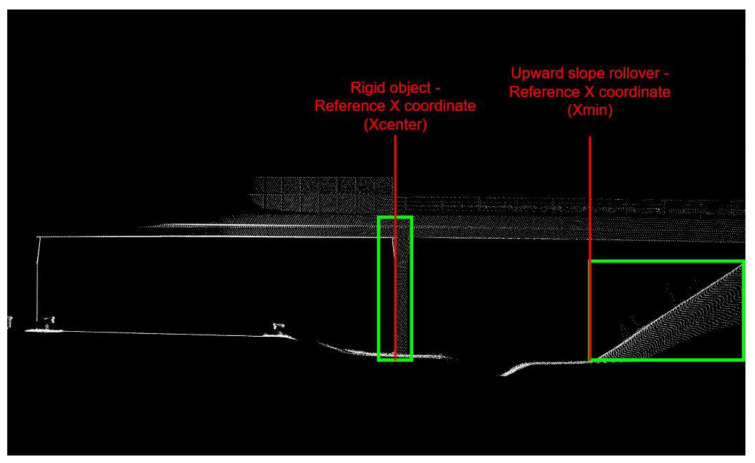
Example of reference X coordinate of rigid object and reference X coordinate of upward slope rollover object.

In terms of object detection evaluation considering 13 iRAP object classes, AP was calculated for each object class as well as mAP. Except for AP and mAP, the confusion matrix is calculated based on the predicted and ground truth objects. Spatially, the RMSE was calculated for the reference X coordinate of each object class. The reference X coordinate for each object class represents the X coordinate of the detected bounding box, which is used to determine the distance between the road edge (Xmax of the road bounding box) and detected bounding box. For the tree, rigid, and semi-rigid object classes, the reference X coordinate is the center of the detected bounding box (Xcenter), while the left edge of the detected bounding box (Xmin) is the reference X coordinate for other object classes. An example of reference X coordinate for the rigid and upward slope rollover classes is shown in Figure 8.

After detecting roadside objects and determining the distance to road edge, it is necessary to code only one object class for the whole 10-m road segment. It is based on the list of roadside hazards from the iRAP Coding Manual [5] (page 50). The list of roadside hazards is based on the object class defined by iRAP and its distance from road edge. After RSS–O is determinated, RSS–D is the distance from the reference X coordinate of RSS–O to road edge. The final values of RSS–O and RSS–D represent the predicted data in the evaluation process of this framework. For the ground truth data, the RSS–O attribute is manually coded for 1951 images in the test dataset. The manual coding is based on the combination of georeferenced video with road cross-section images. The RSS–D is measured manually on road cross-section images. To evaluate the final classification of road segments, the confusion matrix of predicted and ground truth data and other statistical values such as accuracy, precision, and recall are provided in this paper.

## 3. Results

The core part of framework is based on YOLO object detection of the road and iRAP attributes: RSS–O and RSS–D. Therefore, results are divided into object detection evaluation, spatial accuracy of detected objects, and evaluation of road segments classification.

### 3.1. Object Detection Evaluation

**Figure 9:**
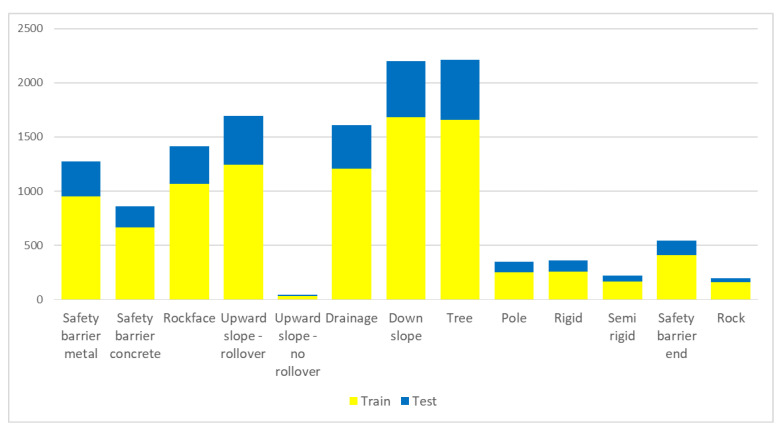
Distribution of labelled RSS–O classes.

Road detection is performed with recall of 0.956, precision of 0.960 and AP 0.949. In terms of RSS–O, 12,987 iRAP defined objects were labeled. Distribution of labeled RSS–O classes is shown in Figure 9.

To present performance of YOLO object detection on RSS–O, confusion matrix, recall, precision and AP are provided for each object. Stated evaluation metrics are based on a test dataset. Confusion matrix is shown in Table 2, while recall, precision and AP are shown in Table 3. Moreover, inference time for one image is 10.1 ms.

### 3.2. Spatial Accuracy of Detected Objects

Spatial accuracy of detected object is presented by RMSE value calculated on the test dataset. The right edge of the road is detected with an RMSE of 0.08 m. RMSE values for every class of detected objects are presented in Table 4.

### 3.3. Evaluation of Road Segments Classification

Evaluation of final classification of road segments into one RSS–O class and one RSS–D class is presented separately by accuracy, precision, recall, and confusion matrix.

Final classification of road segments into one RSS–O is conducted with an accuracy of 85.1%, precision of 0.888, and recall of 0.853. Confusion matrix of road segments classification into RSS–O classes is shown in Table 5. The “None” class in the predicted part of the matrix stands for those road cross sections where none of the iRAP-defined classes are detected.

In terms of road segment classification into one RSS–D class, an accuracy of 85.6%, precision of 0.825, and recall of 0.810 are achieved. Confusion matrix of road segments classification into RSS–D classes is shown in Table 6. Looking at both attributes collectively, 81.1% of all road cross sections in the test dataset are classified correctly by both RSS attributes.

## 4. Discussion

This paper proposes a new framework for determining two road attributes defined by iRAP: RSS–O and RSS–D. Compared to related papers, this work has several improvements in detecting roadside objects and their distance from road edge. First, this framework is based on Lidar data, i.e., point clouds that allow spatial observation. Several authors in related studies [17,18,25,26] have used vehicle-mounted cameras for a similar task and performed roadside object detection from videos. This approach is not suitable for determining the RSS–D attribute because high accuracy in distance determination cannot be achieved. This thesis is supported by the fact that only Jan Z. et al. (2019) [25] attempted to calculate the distance between roadside objects and the road edge from images, but did not evaluate the distance determination. Additionally, using point clouds allows different views to road segments, including the front view, i.e., considering road segments as road cross sections. Apart from detection from videos, there are few studies [22,23,27] that have used Lidar data to determine RSS–O and RSS–D. While Martin-Jimenez et al. (2018) [22] do not focus on distance determination and evaluation of its accuracy, Zhong M. et al. (2019) [23] have evaluated the accuracy of distance determination. They perform the evaluation of distance determination only for two classes: poles and trees. The given evaluation is expressed in terms of average error distance. The average error distance for poles and trees is 0.1 m and 0.5 m, while our average error distance for the same classes is 0.07 m and 0.38 m, respectively. It is obvious that we have achieved much better accuracy in distance determination, especially for the tree class. Moreover, spatial observation allows very easy detection with high accuracy of those iRAP RSS–O classes defined by some spatial parameters such as angles for classes down slope, upward slope—rollover and upward slope—no rollover or width and height for the classes drainage and rock. For example, the down slope class is defined by iRAP as a roadside slope if the slope is less than −15°. The absence of these types of classes in papers [17,18,25,26] suggests that it is not possible to identify these classes from videos. While works based on Lidar data [22,23] did not solve this problem, we proved that classes defined by spatial parameters can be detected from point clouds with high accuracy. Second, except for spatial observation, our framework is fully automated, which is achieved by a high level of inference time. In related works, we do not find any information on inference time. Therefore, our framework is not comparable to similar works in terms of inference time. Moreover, automating the process enables consistent determination of RSS–O and RSS–D classes which is a significant improvement over manual coding of road segments currently used in practice but prone to errors due to [20,23]. Third, the proposed framework includes the recognition of all RSS–O classes existing in Croatia. These are 13 classes, including those defined by spatial parameters. Related works [17,23,25,26] focus on the detection of only a few iRAP-defined classes such as poles, trees, roads, metal guideposts, warning signs, speed signs, etc. The mentioned classes are very easy to detect from images due to the large number of publicly available datasets containing these classes.

In the detection evaluation, we achieved high scores for precision, recall, and AP, i.e., mAP, especially when we consider a large imbalance in the dataset. The class with the highest AP is safety barrier concrete, followed by safety barrier metal (>0.92). The rockface, pole and rock classes have the worst AP (<0.75). Classes with clearly defined boundaries and shapes, such as safety barriers, are detected with high accuracy, while classes whose boundaries and shapes are not clearly defined, such as rockface and rock, are very difficult to detect. An exception is the pole class, which is not precisely defined in iRAP. In the iRAP Manual Guide [5], it is defined as any pole with a diameter greater than 10 cm. This includes everything from light poles to large guideposts. The mentioned objects are not even similar in shape and size, making detection very difficult. The problem can be solved by dividing objects from the pole class to sub-classes (for example: poles, large signposts, small signposts, traffic signs, etc.) and placing them back into the pole class after the detection process. We assume that processing will increase the AP of the pole class defined by iRAP. Despite these obstacles, the final classification of road segments by RSS–O classes is 85.1%.

In terms of spatial evaluation calculated using the RMSE, the safety barrier metal, the safety barrier end and rock classes have the highest accuracy, while the tree and upward slope-rollover classes have low spatial accuracy. It is clear that classes with larger size have larger error, i.e., larger RMSE value, while classes with small size have small RMSE value. The RMSE value is directly related to the RSS–D attribute, which is correctly classified for 85.55% of the road segments.

Apart from all the improvements presented with this framework, there are some drawbacks. First, the price of the Lidar system is still much higher than that of cameras for video-based coding of road segments. There are some low-cost Lidar systems, but their accuracy does not match needs for solving this type of tasks. Considering that [40,41,42] some cars already use Lidar for autonomous driving [43,44,45], there is an intention that it will become cheaper and more affordable. In addition to the price and affordability of Lidar, another potential problem is the size of the dataset. iRAP RSS–O classes are very specific and there are not enough large datasets to help achieve higher accuracy. A large dataset would largely solve the problem of data imbalance, which in turn would solve the problem of many background false negative objects, i.e., unrecognized objects. Unrecognized objects can have a big negative impact on the final accuracy of the whole process, but also on road safety. This work is based on manually annotated objects, but the presence of a larger, balanced dataset would simplify and improve performances of the whole process.

## 5. Conclusions

In this paper, a framework for determining object and distance attributes for iRAP-defined roadsides is proposed. The framework is based on the integration of Lidar point clouds and deep learning-based object detection. It can be divided into two parts: mobile Lidar data collecting with point clouds processing and object detection process on road cross-section images. Compared to standard iRAP coding methods and recent works, this framework allows the spatial consideration of road segments, which is equally important for both RSS–O and RSS–D attributes. In addition, the framework is fully automated with a high level of inference time. It provides consistency in determining both attributes, which is a great improvement over the manual coding of road segments that is common practice today. It also allows road segments to be classified into 1 of the 13 RSS–O classes defined by iRAP, as well as into 1 of the 4 RSS–D classes. Although most of the RSS–O classes are classified with a high AP, some classes have a lower AP value due to class imbalance in the dataset and fuzzy definitions of the classes by iRAP. Nevertheless, the final classification of road segments is achieved by the RSS–O attribute with an accuracy of 85.09%, while the classification accuracy related to the RSS–D attribute is 85.55%. 

There is still much room for progress in the field of automated road infrastructure safety assessments. To improve the classification of the RSS–O attribute, a larger annotated dataset needs to be created to reduce the imbalance between classes and consequently achieve a high level of AP for all classes. Moreover, the augmentation process with an existing dataset can be explored to find out if it will result in the improvement of AP by single class. Furthermore, in addition to these two attributes, iRAP defines another 62 attributes that are necessary for road assessment. There is much room for exploring possible solutions for automating the process of determining other attributes by using different sensors and processing techniques.

## Figures and Tables

**Table 1:** Technical specification of Trimble MX8 Land Mobile Mapping System.

System Module	Parameter	Value
Laser scanning	Accuracy	10 mm
	Precision	5 mm
	Frequency	Variable: 50–300 kHz (×2)
	Range	@50 kHz: 180 m σ ≥ 10%; 500 m σ ≥ 80%
		@300 kHz: 75 m σ ≥ 10%; 200 m σ ≥ 80%
Imaging modules	15 MP Forward panorama	Yes
	15 MP Rear panorama	Optional
	5 MP Oblique Surface	Yes
Positioning		POS LV 420

**Table 2:** Confusion matrix for predicted and ground truth objects presented with percentage.

		Ground Truth													
		Safety Barrier Metal	Safety Barrier Concrete	Rockface	Upward Slope-Rollover	Upward Slone-No Rollover	Drainage	Down Slope	Tree	Pole	Rigid	Semi rigid	Safety Barrier End	Rock	Background False Positive
**Predicted**	**Safety barrier metal**	92.9	0.5										8.3		7.4
	**Safety barrier concrete**		98.0												0.2
	**Rockface**			78.5	3.6										10.7
	**Upward slope-rollover**			10.9	82.7	8.3									14.5
	**Upward slope-no rollover**					83.3									0.2
	**Drainage**						94.3	0.2							12.1
	**Down slope**							81.3							19.9
	**Tree**								90.2						23.5
	**Pole**									75.0					2.4
	**Rigid**					8.3					79.8				1.8
	**Semi rigid**										1.0	89.3			2.6
	**Safety barrier end**	2.8										1.8	88.0		4.0
	**Rock**													75.0	0.6
	**Background False Negative**	4.3	1.5	10.6	13.7	0.0	5.7	18.5	9.8	25.0	19.2	8.9	3.8	25.0	

**Table 3:** Recall, precision, and AP for each class as well as mean recall, precision and AP.

	Recall	Precision	AP
**Safety barrier metal**	0.87	0.92	0.91
**Safety barrier concrete**	0.99	0.98	0.98
**Rockface**	0.81	0.77	0.72
**Upward slope-rollover**	0.77	0.81	0.76
**Upward slope-no rollover**	0.90	0.83	0.83
**Drainage**	0.87	0.93	0.91
**Down slope**	0.78	0.89	0.85
**Tree**	0.81	0.88	0.82
**Pole**	0.87	0.71	0.73
**Rigid**	0.88	0.78	0.76
**Semi rigid**	0.78	0.89	0.87
**Safety barrier end**	0.79	0.88	0.85
**Rock**	0.90	0.75	0.74
**Mean**	0.85	0.85	0.83

**Table 4:** RMSE value for every RSS–O class.

	RMSE (m)
**Safety barrier metal**	0.05
**Safety barrier concrete**	0.06
**Rockface**	0.27
**Upward slope-rollover**	0.65
**Upward slope-no rollover**	0.12
**Drainage**	0.73
**Down slope**	0.36
**Tree**	0.65
**Pole**	0.09
**Rigid**	0.08
**Semi rigid**	0.15
**Safety barrier end**	0.05
**Rock**	0.05
**Mean**	0.25

**Table 5:** Confusion matrix for final road segments classification into one of RSS–O classes presented with percentage.

		Ground Truth												
		Safety Barrier Metal	Safety Barrier Concrete	Rockface	Upward Slope-Rollover	Upward Slone-No Rollover	Drainage	Down Slope	Tree	Pole	Rigid	Semi Rigid	Safety Barrier End	Rock
**Predicted**	**Safety barrier metal**	78.6		0.4	0.6		0.5	1.1	1.3	2.8			2.4	
	**Safety barrier concrete**		93.0	0.0	0.3			1.3	0.6					
	**Rockface**			84.1	5.7			0.2	2.6	1.4	6.1		0.8	20.0
	**Upward slope-rollover**	1.9		8.3	88.1		0.5	0.2	3.9		2.0		0.8	
	**Upward slope-no rollover**					90.0								
	**Drainage**	2.9	0.9	0.7	0.3		85.2	4.2	1.3	1.4				
	**Down slope**	4.9	6.1	0.4			10.1	84.4	8.4	1.4			3.1	
	**Tree**	1.9		1.1	1.8		1.1	5.0	77.3	1.4	6.1		2.4	
	**Pole**	1.9			0.3				0.6	86.1			0.8	
	**Rigid**				0.3	10.0				1.4	81.6			
	**Semi rigid**	1.9									0.0	93.1		
	**Safety barrier end**	5.8		2.2	0.3		1.1	0.7	0.6		2.0	6.9	89.8	
	**Rock**								1.3					77.1
	**None**			2.9	2.4		1.6	2.9	1.9	4.2	2.0			2.9

**Table 6:** Confusion matrix for final road segments classification into one of RSS–D classes presented with percentages.

		Ground Truth			
		0–1 m	1–5 m	5–10 m	>10 m
**Predicted**	**0–1 m**	86.59	3.80	3.76	2.65
	**1–5 m**	9.96	88.43	11.65	15.04
	**5–10 m**	2.03	4.17	78.20	6.19
	**>10 m**	0.81	1.30	4.14	70.80
	**None**	0.61	2.31	2.26	5.31

## Data Availability

The data presented in this study are available on request from the corresponding author. The data are not publicly available due to privacy concern.
